# 10-Methacryloyloxydecyl Dihydrogen Phosphate (10-MDP)-Containing Cleaner Improves Bond Strength to Contaminated Monolithic Zirconia: An In-Vitro Study

**DOI:** 10.3390/ma15031023

**Published:** 2022-01-28

**Authors:** Mohamed M. Awad, Feras Alhalabi, Khaled Mosfer Alzahrani, Majed Almutiri, Fawaz Alqanawi, Lafi Albdiri, Abdullah Alshehri, Ali Alrahlah, Mohammed H. Ahmed

**Affiliations:** 1 Department of Conservative Dental Sciences, College of Dentistry, Prince Sattam Bin Abdulaziz University, Al-Kharj 11942, Saudi Arabia; f.alhalabi@psau.edu.sa (F.A.); am.alshehri@psau.edu.sa (A.A.); 2 Department of Prosthetic Dental Sciences, College of Dentistry, Prince Sattam Bin Abdulaziz University, Al-Kharj 11942, Saudi Arabia; k.alzahrani@psau.edu.sa; 3 College of Dentistry, Prince Sattam Bin Abdulaziz University, Al-Kharj 11942, Saudi Arabia; 434050472@std.psau.edu.sa (M.A.); 435050096@std.psau.edu.sa (F.A.); 435050943@std.psau.edu.sa (L.A.); 4 Department of Restorative Dental Science, College of Dentistry, King Saud University, Riyadh 11545, Saudi Arabia; 5 Engineer Abdullah Bugshan Research Chair for Dental and Oral Rehabilitation, King Saud University, Riyadh 11545, Saudi Arabia; 6 Department of Dental Biomaterials, Faculty of Dentistry, Tanta University, Tanta 31527, Egypt; m.ahmed@dent.tanta.edu.eg

**Keywords:** zirconia, bond strength, contamination, 10-methacryloyloxydecyl dihydrogen phosphate

## Abstract

Contamination of zirconia restorations before cementation can impair the resin–zirconia bonding durability. The objective of this study was to evaluate the effect of human saliva or blood decontamination with 10-methacryloyloxydecyl dihydrogen phosphate (10-MDP)-containing cleaner on the resin–zirconia shear bond strength (SBS). Methods: A total of 220 zirconia specimens were prepared and air-abraded and randomly distributed into 11 groups (*n* = 20 per group). Except for the control group (no contamination), zirconia specimens were contaminated with either human saliva (five groups) or blood (five groups), and then subjected to one of five cleaning methods: water rinsing, 38% phosphoric acid etchant (Pulpdent Corp., Watertown, MA, USA), 70% isopropanol alcohol (Avalon Pharma, Riyadh, Saudi Arabia), Ivoclean (Ivoclar Vivadent, Schaan, Lichtenstein) and Katana Cleaner (Kuraray Noritake, Tokyo, Japan). The resin–zirconia SBS was tested at 24 h and after thermocycling (10 k cycles). Three-way ANOVA followed by Tukey’s multiple comparisons test were utilized to analyze the SBS data. Failure modes were evaluated using a scanning electron microscope. Results: Both blood and saliva significantly affected resin–zirconia SBS as contaminants. After thermocycling, there was no statistically significant difference between SBS obtained after decontamination with the Katana Cleaner (blood, 6.026 ± 2.805 MPa; saliva, 5.206 ± 2.212 MPa) or Ivoclean (blood, 7.08 ± 3.309 MPa; saliva, 6.297 ± 3.083 MPa), and the control group (no contamination, 7.479 ± 3.64 MPa). Adhesive and mixed failures were the most frequent among the tested groups. Conclusion: Both 10-MDP-containing cleaner (Katana Cleaner) and zirconium oxide-containing cleaner (Ivoclean) could eliminate the negative effect of saliva and blood contamination on resin–zirconia SBS.

## 1. Introduction

Zirconium dioxide (zirconia) ceramic is a biocompatible material with excellent mechanical properties and a wide range of clinical applications in esthetic anterior and posterior indirect restorations [1]. Despite their wide applicability in restorative and prosthetic dentistry, the clinical success of zirconia restorations is affected by the durability of the bond strength between zirconia and the tooth structure [2,3]. Zirconia restorations adhesively cemented using resin-based cements exhibit superior clinical performance compared to those cemented with conventional acid-base cements, such as glass ionomer cement [4]. Resin–zirconia bonding usually involves two main interfaces: one is between the tooth structure and the resin-based cement and the second is between the resin-based cement and zirconia [5], which may be a weak adhesive interface, as per clinical results [6]. Air-abrasion of the zirconia surface using aluminum oxide particles followed by the application of 10-methacryloyloxydecyl dihydrogen phosphate (10-MDP)-containing primers are indispensable steps prior to resin cementation of zirconia restorations [7,8]. On the other hand, mechanical treatment of the zirconia surface by air-abrasion results in marked surface topographic changes, increasing the resin–zirconia bond strength [9]. However, air-abrasion using a high air pressure could deteriorate zirconia’s flexural strength and generate structural microcracks [10]. In addition, the chemical reaction between 10-MDP-based primers and zirconia is reported to enhance resin–zirconia bonding [11]. These 10-MDP-containing primers could produce strongly adsorbed films on the zirconia surface, with evidence of phosphate salt formation [12]. Moreover, the cementation of zirconia restorations with 10-MDP-based resin cements results in higher bond strength [5].

The zirconia bonding and testing conditions applied in laboratory studies are ideal, but in certain clinical situations, contamination of zirconia restorations by saliva and/or blood may occur during the try-in step [13]. This contamination can impair the chemical reaction between 10-MDP-based primers and zirconia surface and can consequently decrease the durability of the resin–zirconia bonding [14,15,16].

Since water rinsing is ineffective for zirconia decontamination [17], several decontamination methods have been attempted. Air-abrasion of zirconia is one of the effective decontamination methods used to eliminate different contaminants [14,15]. However, air-abrasion used in the decontamination process of zirconia has been reported to cause intrinsic phase changes within zirconia [18,19]. Non-thermal atmospheric plasma has been successfully applied to eliminate saliva contamination on zirconia surfaces [20,21], however, it might not be familiar to many dental practitioners because of its difficult clinical applicability. Clinically relevant materials such as the phosphoric acid etchant or alcohol were also tested in vitro as decontaminants for zirconia; however, both showed controversial results [15]. Recently, a zirconium oxide-containing cleaning solution designed for decontamination of indirect restorations has shown promising results for saliva [17,20] and blood [17] decontamination. Despite its proven cleaning effectiveness, it is not indicated for intra-oral cleaning. Katana Cleaner (Kuraray Noritake, Japan) has recently been introduced as the first 10-MDP-based cleaner for cleaning contaminated dental substrates such as zirconia, both intra-orally and extra-orally.

A recent study investigated the effect of a 10-MDP-containing cleaner on the bond strength saliva-contaminated zirconia [22], and although promising results were obtained, artificial aging or thermocycling were not included in evaluating the bonding durability. Therefore, the objective of this investigation is to evaluate the effect of saliva and blood decontamination on resin–zirconia bonding. The null hypotheses are: (1) There would be no significant effect of saliva and blood contamination on resin–zirconia shear bond strength (SBS). (2) The resin–zirconia SBS after decontamination would not be significantly different irrespective of the decontamination method applied.

## 2. Materials and Methods

The materials used in the study and their compositions are described in Table 1.

### 2.1. Zirconia Specimen Preparation

Pre-sintered zirconia blocks (inCoris TZI C, Sirona Dental Systems GmbH, Bensheim, Germany) were milled into a total of 220 smaller blocks with the dimensions of 8 mm length, 8 mm width and 3 mm height using a dental milling machine (CAM 5-S1, vhf camfacture AG, Ammerbuch, Germany). The prepared zirconia blocks were sintered according to the manufacturer’s instructions and embedded in a self-curing acrylic resin. The top surface of zirconia specimens was polished for 2 min with 600-grit silicon carbide paper mounted on a polishing machine at a speed of 300 rpm under a water coolant and subjected to air-abrasion using 50 μm Al_2_O_3_ with 2 bar air pressure for 20 s according to the manufacturer instructions, followed by ultrasonic cleaning in distilled water for 5 min, and subsequently air-dried.

### 2.2. Contamination and Decontamination

Under the Institutional Review Board (IRB) approval (PSAU2020028) obtained from the College of Dentistry, Prince Sattam Bin Abdulaziz University, a total of 10 mL of saliva and 10 mL of venous blood were obtained from a healthy donor (one investigator), collected in plastic tubes, and used within 24 h. Zirconia specimens were randomly divided into 11 groups (*n* = 20 per group) according to the contamination and decontamination protocols detailed in Table 2. Except for the uncontaminated group (Un-No), zirconia surfaces of all groups were covered by human saliva or human blood for 1 min, followed by the cleaning protocols (steps) clearly described in Table 2. All specimens were gently air-dried before the priming step.

### 2.3. Shear Bond Strength (SBS) Test

Zirconia-primer (Z-Prime Plus; Bisco Inc., Schaumburg, IL, USA) was applied to specimens of each group and air-dried for 5 s according to the manufacturer’s instructions. Then, a silicon mold with the dimensions of 3 mm diameter and 2 mm height was fitted on the zirconia surface and filled with a dual-cure resin cement (Multilink N; Ivoclar Vivadent, Schaan, Liechtenstein). The resin cement was subsequently light-cured for 40 s to form the resin build-up. One half of the bonded specimens of each group were stored in distilled water at 37 °C for 24 h; thus, adequate polymerization of the dual-cured resin cement was achieved before the evaluation of resin–zirconia SBS, while the other half of specimens were tested after being subjected to thermocycling (10 k cycles) as a form of artificial aging before SBS testing. Each cycle involved immersion of the specimens in distilled water with a temperature of 5 and 55 °C for 30 s each, while the transfer time was 5 s.

**Figure 1:**
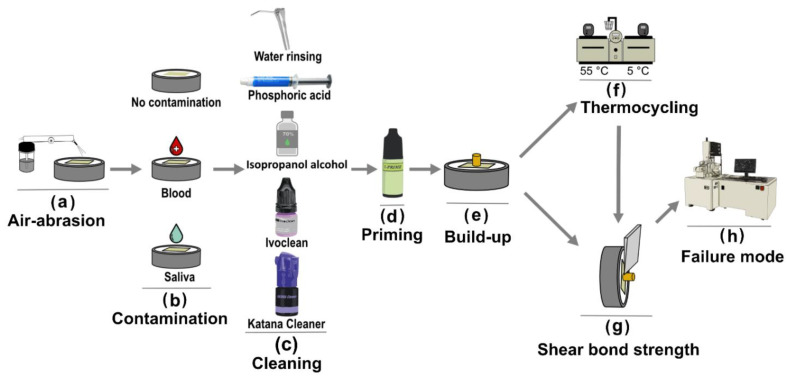
Schematic illustration of the study design showing variables employed in specimens’ preparation. (**a**) Air-abrasion of zirconia ceramic employed to all specimens. (**b**) Contamination: no contamination or contaminated specimens (blood or saliva). (**c**) Cleaning (decontamination) methods (water rinsing ‘no cleaner’, 38% phosphoric acid etching, 70% isopropanol alcohol, Ivoclean (Ivoclar Vivadent) or Katana Cleaner (Kuraray Noritake)). (**d**) Zirconia priming. (**e**) Resin cement build-up. (**f**) Thermocycling (10 k TC). (**g**) SBS testing. (**h**) Failure mode evaluation using scanning electron microscope (SEM).

**Figure 2:**
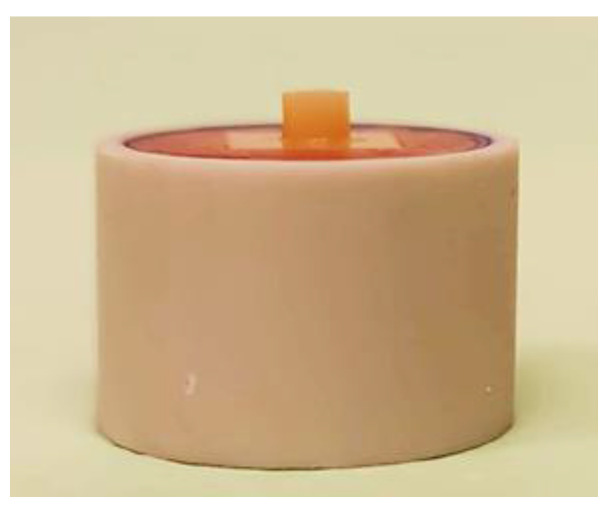
Representative image of a bonded specimen before testing.

The cross-sectional diameter of the bonded interface was measured with a digital caliber. A metal chisel mounted on a universal testing machine (Instron 5965, Instron Corporation, Norwood, MN, USA) was utilized to apply a shear force perpendicular to the resin–zirconia adhesive interface at a cross speed of 0.5 mm/min until failure or debonding. SBS in MPa was calculated by dividing the force recorded at failure or debonding in Newton (N) by the cross-sectional area of the bonded interface in mm^2^. The study design and specimens’ preparation steps are illustrated in Figure 1. A representative image of a bonded specimen before testing is provided in Figure 2.

### 2.4. Failure Mode Evaluation

Failure modes were evaluated using a scanning electron microscope (SEM) (JSM-6610LV, JEOL Ltd., Tokyo, Japan) at ×25 magnification after gold sputtering using a sputter coater (fine coat ion sputter JFC1100, JEOL Ltd., Tokyo, Japan). Failure modes were classified into adhesive interfacial failure, cohesive failure in resin cement, cohesive failure in zirconia and mixed failure, which involved adhesive and cohesive failures.

### 2.5. Statistical Analysis

Three-way analysis of variance (ANOVA) was used to statistically analyze the SBS data of all tested groups for overall significance, evaluating the effect of contamination (saliva or blood), cleaning method (water rinsing ‘no cleaner’, 38% phosphoric acid etching, immersion in 70% isopropanol alcohol, Ivoclean (Ivoclar Vivadent) and Katana Cleaner (Kuraray Noritake)), artificial aging (24 h or TC) and their interactions on resin–zirconia SBS. The difference between specific groups’ means was statistically analyzed with Tukey’s multiple comparisons test (*p* < 0.05).

## 3. Results

### 3.1. SBS

**Figure 3:**
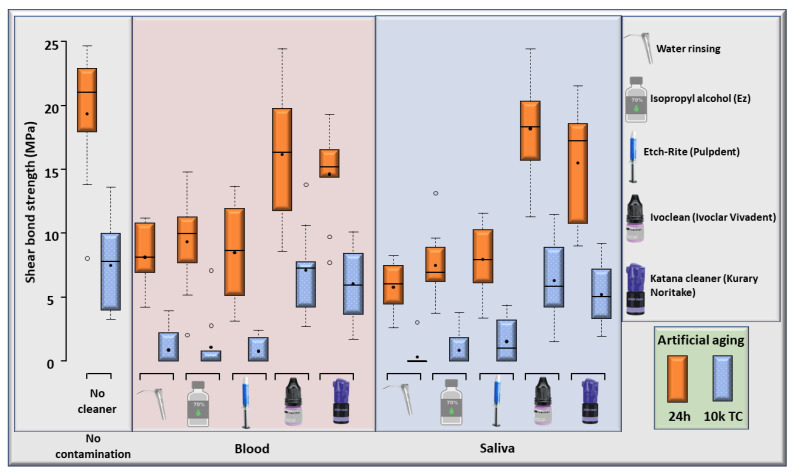
Box–whisker plots of the resin–zirconia shear bond strength (SBS) (in MPa) of all experimental groups, either measured at 24 h (24 h) or after artificial aging with 10 k thermocycles (10 k TC). The black closed dots represent the mean SBS. The horizontal line within each box represents the median SBS.

SBS means and standard deviations (SDs) of all experimental groups, at 24 h and after 10 k TC, are graphically illustrated in Figure 3. Moreover, Table 3 provides a detailed numerical description of SBS, ptf’s (pre-test failures) recorded for each group and the statistical difference within each group at 24 h and after 10 k TC.

In the first step of the statistical analysis, a first-order interaction for each of the three study variables: ‘Contamination’, ‘Cleaner’, and ‘Aging’, was individually tested. All the three study variables were found significant, indicating that each individual variable should have influenced the resulted SBS. Second-order interaction analysis revealed that the ‘Contamination × Cleaner’ interaction was statistically non-significant, indicating that the effect of the cleaner (decontamination method) should not have been affected by changing the type of contamination, on the resultant SBS. However, the other two second-order interactions were found significant, meaning that the resulted SBS would have been affected by changing the cleaner and the type of contamination. The third-order interaction, combining all three variables, was found non-significant and hence proposed as the final statistical model, based upon which data was analyzed using Tukey post-hoc testing analysis to accurately detect the individual differences between the experimental groups (Table 3).

Both saliva and blood contamination significantly (*p* < 0.001) reduced the resin–zirconia SBS, with no statistically significant difference between them at 24 h and after thermocycling. The SBS means following decontamination of zirconia using phosphoric acid etchant or alcohol were significantly (*p* < 0.001) less than those of uncontaminated groups, with no statistically significant difference compared to water rinsing. The SBS means following decontamination of zirconia using the zirconium oxide-based cleaner (Ivoclean) and 10-MDP-based cleaner (Katana Cleaner) were statistically comparable to those of the uncontaminated group, with no significant difference between the two cleaning methods, at 24 h and after 10 k thermocycling. Thermocycling (10 k TC) significantly affected the resin–zirconia SBS of all groups. The results of the three-way ANOVA are described in Table 4.

### 3.2. Failure Modes

**Figure 4:**
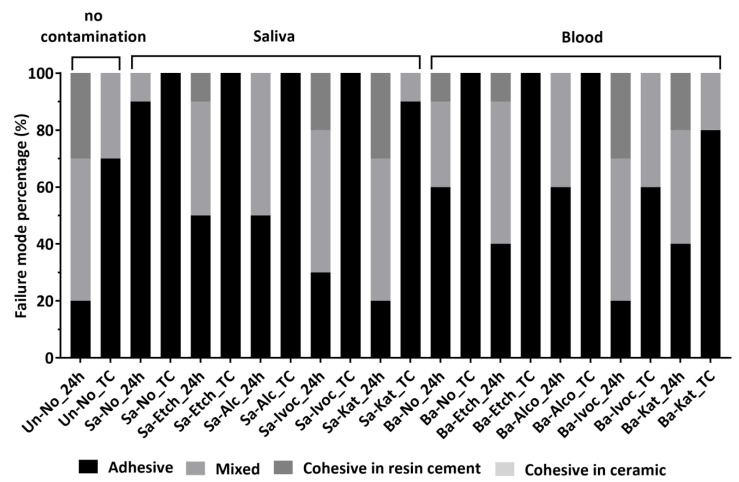
The frequencies of failure modes (expressed as percentages) recorded for all shear bond strength tested groups (Un: uncontaminated; No: water rinsing (no cleaner); Sa: saliva; Etch: phosphoric acid etching; Alc: 70% isopropanol alcohol; Ivoc: Ivoclean (Ivoclar Vivadent); Katana Cleaner (Kuraray Noritake) at 24 h and after thermocycling (10 k TC). The incidence of adhesive and mixed failures was the most common in saliva- and blood-contaminated groups.

**Figure 5:**
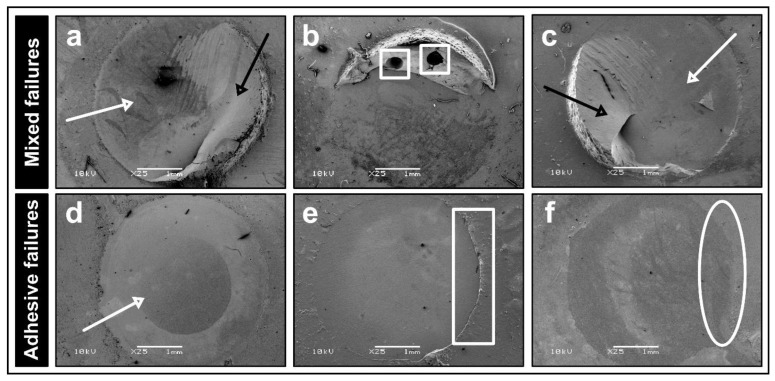
Representative SEM micrographs of mixed (**a**–**c**) and adhesive (**d**–**f**) failure modes at ×25 magnification. Mixed failures included adhesive failure at the resin–ceramic interface (white arrow, **a**,**c**) and cohesive failure within the resin cement (black arrow, **a**,**c**). Voids (white square, **b**) were noticed within the resin cement. Adhesive failures involved interfacial debonding between resin cement and ceramic with no remaining resin cement at the ceramic surface (white arrow, **d**). While the remaining zirconia primer can be detected (white rectangle, **e**) after 24 h of testing, it was washed away (white ellipse, **f**) after thermocycling.

The frequencies of the observed failure modes in the respective groups are illustrated in Figure 4. Adhesive failures (Figure 5d–f) were the most frequent in both no decontamination groups (Sa-No, Bl-No) at 24 h and after 10 k thermocycling. The mixed failures (Figure 5a–c) were the most predominant in the other groups at 24 h, while after thermocycling, Sa-Etch, Sa-Alc, Bl-Etch and Bl-Alc groups presented high percentages of adhesive as well as pre-test failures. No cohesive failures in ceramic (zirconia) substrate were recorded in all groups.

## 4. Discussion

The clinical performance of ceramic restorations can be affected by the durability of resin–ceramic bonding [2,23]. Adhesively cemented zirconia restorations are subjected to both shear and tensile forces during chewing [5]. This study was designed to evaluate the effect of decontamination protocols on the resin–zirconia SBS. Despite that the SBS test may be associated with a less uniform stress distribution at the interface compared to the tensile bond strength test, it can provide an adequate ranking of the tested experimental groups if correctly performed and interpreted using fractography analysis. The SBS test is usually utilized to assess the bond strength to different types of dental ceramic materials [24,25]. The surface-treated (air-abraded) zirconia surfaces may be more receptive to contamination owing to the higher surface roughness.

Both saliva and blood contamination significantly (*p* < 0.05) reduced the resin–zirconia SBS, at 24 h and after thermocycling, with no statistically significant difference between them. Therefore, the first null hypothesis was accepted. Rinsing with water or immersion in alcohol cannot eliminate the organic elements such as carbon or nitrogen retained on blood- or saliva-contaminated zirconia surfaces [17]. This may explain the negative impact of the contaminants on the SBS because such organic residue from saliva or blood could impede the chemical reaction between the 10-MDP-primer and zirconia surface, thus decreasing the obtained resin–zirconia SBS [16]. The chemical bonding with the zirconia surface can influence the resin–zirconia bond strength [12]. The chemical affinity between the 10-MDP-primer and zirconia depends on the ability of the phosphate group in the 10-MDP molecule to chemically react with zirconium oxide to form the chemically stable zirconium phosphate [26], which would improve resin–zirconia bonding because zirconium phosphate salts can withstand thermal and hydrolytic degradation and can thus increase the bonding durability [26]. One more advantage of the 10-MDP-primer is the methacrylate group because it enables copolymerization with methacrylate-based materials such as resin cements, which has been confirmed via the chemical analysis of the zirconia primed surface [12]. In addition, the methacrylate-functionalized phosphate monomers, such as the 10-MDP molecule, can decrease the potential for transformation of the tetragonal to monoclinic zirconia [27], which would help in maintaining the strength of zirconia over time. In contrast to alcohol cleaning, the application of the phosphoric acid etchant can partially eliminate saliva contamination from zirconia surfaces [14,28]. However, phosphoric acid cleaning did not improve resin–zirconia SBS in this study. In fact, phosphoric acid etching can decrease the surface energy of zirconia surfaces [14]. In addition, it is reported that phosphoric acid can react with zirconia, resulting in leaving a phosphorous residue on the zirconia surface, which would negatively affect the chemical reaction between 10-MDP molecules and zirconia and thus reduce the resin–zirconia bond strength [16,29]. It is reported that the use of phosphoric acid for cleaning of contaminated zirconia impairs resin–zirconia bonding [22].

Zirconia decontamination with Ivoclean and Katana Cleaner outperformed decontamination with phosphoric acid or alcohol in terms of shear bond strength, at 24 h and after artificial aging by thermocycling. Thus, the second null hypothesis that there is no difference between the effect of the decontamination protocols tested cannot be accepted. Furthermore, unlike the cleaning or decontamination methods utilized in this study, the use of Ivoclean and Katana Cleaner resulted in SBS means similar to that of the control (uncontaminated) group. Ivoclean is composed of a hyper-saturated solution of zirconia particles. Upon application of Ivoclean onto contaminated substrates, it adsorbs contaminants such as phosphate; in this way, contaminants can be removed from the surface of the contaminated ceramic surface. Previous X-ray photoelectron spectroscopy (XPS) analysis proved the efficacy of Ivoclean as a ceramic cleaner as it eliminates the residue of organic contaminants such as saliva or blood [17,30,31]. Moreover, Ivoclean can increase the surface energy of the decontaminated ceramic surface [30]. This can explain why the bond strength between the decontaminated, cleaned ceramic surface and Ivoclean did not significantly differ from that between the uncontaminated surface and Ivoclean [30,31].

The SBS statistical analysis of this study revealed that there was no statistically significant difference between Ivoclean and Katana Cleaner, at 24 h and after artificial aging by thermocycling, which is in accordance with a recent study in which the effect of the two ceramic cleaners on resin–zirconia SBS was evaluated [22]. The 10-MDP salt is the active ingredient of Katana Cleaner which approaches the organic residue (contamination) during the application of the cleaning solution with rubbing; then, the hydrophobic group of the 10-MDP salts attaches to and surrounds the contaminant, which is subsequently washed away upon being rinsed with water [32,33]. In addition, a 10-MDP salt cleaner has been used effectively for elimination of temporary cement residue [33], sealer remnants from dentin surface [34] and salivary contaminants on zirconia surfaces [22], thus improving the resin–zirconia bond strength. Two 10-MDP-based experimental cleaning agents with compositions similar to that of Katana Cleaner were effective in eliminating saliva contamination on the zirconia surface, increasing zirconia surface wettability and enhancing the resin–zirconia tensile bond strength [35].

The resin–zirconia SBS of all tested groups was significantly reduced after thermocycling (10 k TC). Thermocycling deteriorates the bond strength of the two adhesively bonded materials [36]. The temperature change creates stress at the adhesive interface due to the different coefficients of thermal expansion of the two adhesively bonded materials (resin cement and zirconia), thus weakening the adhesive interface and decreasing the resin–zirconia bond strength [37]. The reduced resin–zirconia bond strength may result in clinical debonding of zirconia restorations cemented to less-retentive preparations.

The predominant adhesive failures in the groups decontaminated with phosphoric acid etching or alcohol may be an indicator of the decrease in the SBS. Mixed failures in the groups decontaminated using Ivoclean or Katana Cleaner may represent the relatively high bond strength achieved. No cohesive failures in zirconia ceramics were recorded in any group, and this can be explained by the high mechanical properties of sintered zirconia [38]. Cohesive failures in the resin cement may be a result of uneven stress distribution during the shear bond strength testing [39] or due to manipulative errors such as voids existing within the resin cement build-ups (Figure 5b). The pre-test failures occurred only after thermocycling, which may be a sign of reduced bond durability [40] after TC, especially for groups in which the decontamination was performed with phosphoric acid etchant, alcohol or only with water rinsing. The lack of XPS analysis, which would enable a more in-depth evaluation of Katana Cleaner, is one of the limitations of this study.

## 5. Conclusions

This study tested the cleaning effect of human saliva and blood on resin–zirconia SBS. Both zirconium oxide-containing (Ivoclean) and 10-MDP-containing (Katana Cleaner) cleaners were able to overcome the negative effects of saliva and blood contamination on resin–zirconia SBS. There was no statistically significant difference in the resin–zirconia SBS means obtained following decontamination using these two types of cleaning agents. The use of phosphoric acid etchant or alcohol cannot be recommended for cleaning blood- and/or saliva-contaminated zirconia restorations. Both blood and saliva contamination can result in a significant reduction in the resin–zirconia SBS.

## Figures and Tables

**Table 1:** Materials used in the study:

Material (Manufacturer)	Composition
inCoris TZI C medi S (Sirona Dental Systems GmbH, Bensheim, Germany) LOT: 3314000419	ZrO_2_, HfO_2_, Y_2_O_3_, Al_2_O_3_, other oxides
Etch-Rite (Pulpdent Corp., Watertown, MA, USA) LOT: 200114	38% phosphoric acid gel
Isopropanol Alcohol Ez CLEAN (Avalon Pharma; Riyadh, Saudi Arabia. LOT: 2040027	Isopropanol alcohol (C_3_H_8_O, 70%), water (30%)
Ivoclean (Ivoclar Vivadent, Schaan, Lichtenstein) LOT: Y36272	ZrO_2_, water, polyethylene glycol, sodium hydroxide, pigments and additives
Katana Cleaner (Kuraray Noritake, Tokyo, Japan) LOT: 3C0006	Water, 10-MDP, triethanolamine, polyethylene glycol, stabilizer, dyes
Z-Prime Plus (Bisco Inc., Schaumburg, IL, USA) LOT: 2000006418	10-MDP, BPDM, HEMA, ethanol
Multilink N (Ivoclar Vivadent, Schaan, Liechtenstein) LOT: Z00RKM	Dimethacrylate, HEMA, barium glass, ytterbium trifluoride, spheroid mixed oxide

**Table 2:** Contamination and decontamination protocols (groups).

Group Code	Contamination	Decontamination Protocol
Un-No	Uncontaminated	No decontamination
Sa-No	Saliva	Thorough water rinsing for 20 s
Sa-Etch	Saliva	38% Phosphoric acid etching for 20 s Thorough water rinsing for 20 s
Sa-Alc	Saliva	Immersion in 70 % isopropanol alcohol for 2 min Thorough water rinsing for 20 s
Sa-Ivoc	Saliva	Application of Ivoclean (Ivoclar Vivadent) with a micro-brush to cover the bonded area (allow 20 s for the cleaning) Thorough water rinsing until the color of the cleaner disappears
Sa-Kat	Saliva	Application of Katana Cleaner (Kuraray Noritake) with a micro-brush to cover the bonded area Rubbing for at least 10 s Thorough water rinsing until the color of the cleaner disappears
Bl-No	Blood	Thorough water rinsing for 20 s
Bl-Etch	Blood	38% phosphoric acid etching for 20 s Thorough water rinsing for 20 s
Bl-Alc	Blood	Immersion in 70% isopropanol alcohol for 2 min Thorough water rinsing for 20 s
Bl-Ivoc	Blood	Application of Ivoclean (Ivoclar Vivadent) with a micro-brush to cover the bonded area (allow 20 s for the cleaning) Thorough water rinsing until the color of the cleaner disappears
Bl-Kat	Blood	Application of Katana Cleaner (Kuraray Noritake) with a micro-brush to cover the bonded area Rubbing for at least 10 s Thorough water rinsing until the color of the cleaner disappears

**Table 3:** Mean ± standard deviation (SD) shear bond strength (SBS) expressed in mega Pascal (MPa) for the different contamination and decontamination groups at 24 h and after thermocycling (TC).

	SBS (24 h)		SBS (10 k TC)	
	Mean ± ^1^ SD (^2^ MPa)	^3^ ptf/^4^ *n*	Mean ± SD (MPa)	ptf/*n*
**Un-No**	19.33 ± 5.136 ^a^	0/10	7.479 ± 3.64 ^A,^*	0/10
**Sa-No**	5.751 ± 1.874 ^b^	0/10	0.3036 ± 0.96 ^B,^*	9/10
**Sa-Etch**	7.93 ± 2.527 ^b^	0/10	1.508 ± 1.713 ^B,^*	5/10
**Sa-Alc**	7.469 ± 2.711 ^b^	0/10	0.8409 ± 1.435 ^B,^*	7/10
**Sa-Ivoc**	18.18 ± 3.969 ^c^	0/10	6.297 ± 3.083 ^C,^*	0/10
**Sa-Kat**	15.51 ± 4.44 ^c^	0/10	5.206 ± 2.212 ^C,^*	0/10
**Bl-No**	8.117 ± 2.321 ^b^	0/10	0.8493 ± 1.439 ^B,^*	7/10
**Bl-Etch**	8.482 ± 3.999 ^b^	0/10	0.7488 ± 1.041 ^B,^*	6/10
**Bl-Alc**	9.312 ± 3.761 ^b^	0/10	1.061 ± 2.294 ^B,^*	7/10
**Bl-Ivoc**	16.17 ± 5.43 ^c^	0/10	7.08 ± 3.309 ^C,^*	0/10
**Bl-Kat**	14.62 ± 3.439 ^c^	0/10	6.026 ± 2.805 ^C,^*	0/10

^1^: Standard deviation, ^2^: Mega Pascal, ^3^: Pre-test failure, ^4^: Number of specimens per group. Different lower- and upper-case superscript letters indicate significant differences between the experimental groups at 24 h and after 10 k TC, respectively. *: Indicates significant difference for each group before and after artificial aging within each row.

**Table 4:** Three-way ANOVA statistical analysis including first-, second-, and third-order interactions.

	^1^ Df	^2^ Sum Sq	^3^ Mean Sq	F Value	*p*-Value	Significance
**Contamination**	2	734.6	367.3	372,831	<0.001	*
**Cleaner**	4	2274.4	568.6	577,185	<0.001	*
**Aging**	1	3971.5	3971.5	4,031,383	<0.001	*
**Contamination × Cleaner**	4	29.8	7.4	0.7550	0.556	
**Contamination × Aging**	2	61.8	30.9	31,381	0.045	*
**Cleaner × Aging**	4	120.2	30.1	30,509	0.018	*
**Contamination × Cleaner × Aging**	4	46.1	11.5	11,687	0.326	
**Residuals**	198	1950.6	9.9			

^1^ Degrees of freedom, ^2^ Sum of squares, ^3^ Mean square. *: Indicates a statistically significant effect.

## Data Availability

The data presented in this study are available upon request from the corresponding authors.
